# Prescriptions

**Published:** 1896-06

**Authors:** 


MALARIAL HEMATURIA.

Keeping the bowels open with calomel followed by salts, Dr. J. E. Long, of Abbeville, Alabama, uses hot mustard baths and administers the following combinations in alternation every three hours:

℞. Spirit turpent.......... 2 drs.
Acidi carbol.............10 grs.
Pot. chlorat.............. 3 drs.
Spirit lav. comp ........ 2 drs.
Acacia gum.... ....... 3 drs.
Aqua. menth. pep., q. s aa 4 ozs.
M. Sig.: Teaspoonful every three hours.—*Louisville Medical Monthly.*

℞. Fld. Ext. Ergot .........℥ss.

Gallic Acid.............. gr. xl.

Acid Sulphuric (dil.).....ʒ i.

Syr. Ginger. .............

Aqua, aa, q. s..............℥ij.

M. Shake well. Teaspoonful in water every four hours.—*Louisville Medical Monthly.*

PURULENT CONJUNCTIVITIS.

℞. Hydrastis Sulp.
Acid boric
Sodii bi-borat .......aa grs. v.
Tr. opii deodorata...... ℥ ss.
Aqua distillata .........℥ j.
M. Sig. Mix and filte.r. Drop in eye.—*Charlotte Medical Journal.*

DIARRHŒA.

℞. Bismuthi salicylat 8.0, 123½grs.

Naphthol *B*.......8.0, 123½grs.

Pulv. ipecac et opii 4.0, 62 grs.

Acidi tannici......3.0, 46⅓grs

M, Mix and divide in twenty capsules.

Sig.:—One every three hours.

Also:

℞. Acidi sulphurici, 20.0 35 mins.
M. Sig.: Five drops for each glassful of sweetened water.

VOMITING OF PREGNANCY.

For obstinate vomiting during early pregnancy Dr Baer recommends the following:

℞. Bismuth subnitrate.......ʒij.

Saccharated pepsin........ʒj.

Sodium bicarbonate.......ʒss.

Sugar of milk.............ʒj.

M. Mix and make 12 powders.

Sig.: One powder every three hours.

In addition to the above the following prescription will be found most pleasing and effective:

℞. Diluted nitrohydrochloric Acid. .............fʒiss.
Syrup of lemon..........ʒi.
Simple syrup.............℥ij.
M. Sig.:—Give 1 teaspoonful in a wineglass of ice-water three times a day.—*Philadelphia Polyclinic.*

